# Graphene Mesh for Self‐Sensing Ionic Soft Actuator Inspired from Mechanoreceptors in Human Body

**DOI:** 10.1002/advs.201901711

**Published:** 2019-10-11

**Authors:** Rassoul Tabassian, Van Hiep Nguyen, Sima Umrao, Manmatha Mahato, Jaehwan Kim, Maurizio Porfiri, Il‐Kwon Oh

**Affiliations:** ^1^ Creative Research Initiative Center for Functionally Antagonistic Nano‐Engineering Department of Mechanical Engineering Korea Advanced Institute of Science and Technology 291 Daehak‐ro, Yuseong‐gu Daejeon 34141 Republic of Korea; ^2^ Department of Mechanical and Aerospace Engineering Tandon School of Engineering New York University 6 MetroTech Center Brooklyn NY 11201 USA; ^3^ Department of Biomedical Engineering Tandon School of Engineering New York University 6 MetroTech Center Brooklyn NY 11201 USA

**Keywords:** graphene mesh, mechanoreceptors, porous electrodes, self‐sensing, sensory actuators

## Abstract

Here, inspired by mechanoreceptors in the human body, a self‐sensing ionic soft actuator is developed that precisely senses the bending motions during actuating utilizing a 3D graphene mesh electrode. The graphene mesh electrode has the permeability of mobile ions inside the ionic exchangeable polymer and shows low electrical resistance of 6.25 Ω Sq^−1^, maintaining high electrical conductivity in large bending deformations of 180°. In this sensing system, the graphene woven mesh is embedded inside ionic polymer membrane to interact with mobile ions and to trace their movements. The migration of mobile ions inside the membrane induces an electrical signal on the mesh and provides the information regarding ion distribution, which is proven to be highly correlated with the bending deformation of the actuator. Using this integrated self‐sensing system, the responses of an ionic actuator to various input stimulations are precisely estimated for both direct current and alternating current inputs. Even though the generated displacement is extremely small around 300 µm at very low driving voltage of 0.1 V, high level accuracy (96%) of estimated deformations could be achieved using the self‐sensing actuator system.

## Graphical abstract

A graphene mesh electrode having high ion permeability and electrical conductivity is adopted as a self‐sensing ionic soft actuator inspired by mechanoreceptors in the human body. The graphene mesh electrode provides electrical signals sensitive to ion migration inside the polymer electrolyte without blocking their movements and is used to predict the bending deflection of the actuator.

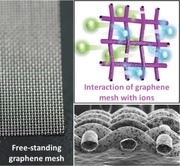

## 1 Introduction

Recent advances in material science and nanotechnology have fueled large investments in the design and fabrication of new electroactive soft devices with stretchable, flexible, and foldable functionalities.^1^ Ionic soft actuators that can be integrated in soft electronic devices have drawn tremendous attention over the last decade. The reason behind their appeal is the generation of high bending deformation under very low input voltage (less than 0.5 V).^2^ As reflected by their name, the actuation mechanism of ionic soft actuators is mainly based on the migration of unequally sized mobile ions inside an electrolyte membrane, resulting in bending deformation under an electric field. Accordingly, most of the research in this area has been devoted to design and fabrication of new electrodes, aiming at improving the bending deflection of the actuators.^3^ Owing to numerous studies in recent years, bending deflections of several millimeters have been achieved through the application of low input stimuli on the order of 0.5 V.^2^, ^4^ In addition to large bending deformation, stronger actuators can be designed to elicit noticeable mechanical forces.^5^

In spite of these promising characteristics, ionic soft actuators possess some imperfections that often preclude their usage in real engineering applications. The main drawback of ionic soft actuators is their unpredictable responses and highly nonlinear behavior.^6^ The majority of engineering applications demand accurate control systems capable of adjusting the movement of components in specific circumstances. The unavoidable nonlinear response of ionic actuators severely challenges the prediction of their behavior, such that the synthesis of effective control systems for this class of actuators remains an elusive issue. Therefore, the development of an efficient sensing method to monitor the bending of ionic soft actuators would constitute a major advancement to address this issue.

In fundamental studies, the mechanical displacement of ionic actuators is usually measured through external sensors, such as laser sensors or strain gauges. However, parallel implementation of external sensors with an actuator is not possible in real applications because of cost and space limitations. Accordingly, some research groups are focusing on the fabrication of self‐sensing actuators, in which sensing and actuation are integrated within a single component for real‐time monitoring of the actuator movement. Most of the currently available mechanisms of self‐sensing actuators require the simultaneous measurement of the electrical resistance along the actuator electrode during actuation. When the actuator is stimulated, one of the electrodes will be stretched while the other is compressed. Since the electrical resistance varies with the effective length of the electrodes, one is able to measure the bending deformation in the form of curvature or tip displacement.

However, inferring the resistance values by measuring the voltage drop along an electrically active component is an intricate task.^7^ Punning et al. proposed a semi‐fixed ionic polymer metal composite (IPMC) actuator with wiring at both ends, which is electrically excited through a signal applied at its mid‐span.^8^ The authors measured the voltage drop along the electrodes in the movable section of the actuator and compared it with the voltage drop along the fixed section to quantify the resistance change, thereby estimating the curvature of the actuator. Due to unavoidable differences in the physical and geometric properties of the fixed and moving sections, the accuracy of the indirect curvature measurement was limited. Another method for measuring the variation of the resistance during actuation entails the use of patterned electrodes. In this method, the electrode is segmented into separate sensing and actuating parts.^9^ A piezo‐resistive sensing signal is acquired from the segments that are not linked to the activated parts of the electrode. Although the sensing and actuating segments are not connected, the cross‐talk effect between adjacent segments constitutes a serious issue that could hinder the performance of the approach.^7^ Size incompetency is another issue of this type of self‐sensing actuator. Segmenting the electrode into sensing and actuating parts enlarges the width of the actuator at least three times. In addition, this type of segmented electrodes requires connection of two extra wires for collecting sensing signal, eventually resulting in the size increase of the actuator and demolishing its capability for being used in tiny systems. Developing novel sensors that are highly sensitive to the movement of mobile ions inside the ionic polymer and electro‐chemo‐mechanical deformations of the actuator, can potentially resolve some of the existing issues of the conventional self‐sensing actuators.

In principle, various kinds of mechanoreceptors in the human body work by changing the ion equilibrium in receptor cells.^10^ When an external pressure is imposed on the human skin, the shape of mechanoreceptors inside the dermis changes. Such a shape change transmits the pressure to the tip of an embedded sensory neurons and deforms their plasma membrane. In turn, the deformation of the plasma membrane opens the ion channels, allowing sodium ions to leak into the cell. The leakage of these ions modulates the electric potential inside the cell, thereby triggering neuron firing. Inspired by the concept of ionic sensing in human's mechanoreceptor, we attempted to design a sensing mechanism that measures the variation of ions' concentration through the thickness of the electrolyte membrane in an ionic actuator. Since bending deformation is mediated by ions motion, the acquisition of data regarding the distribution of ions inside the membrane opens a new pathway for the estimation of the actuator deflection. To afford such a measurement, an ion‐permeable perforated electrode through which mobile ions can migrate should be embedded within the electrolyte membrane to collect electrical signals corresponding to ion movements.

A 3D graphene woven mesh is a perfect candidate for the design of an ion‐permeable sensing electrode due to its liquid permeability and high electrical conductivity. An established way to monolithically assemble graphene woven mesh at a large scale is to grow graphene by chemical vapor deposition (CVD) method on metallic meshes.^11^ Unlike chemically exfoliated graphene, CVD grown graphene specimens possess low levels of defects and enhanced bonding with adjacent graphene sheets in the network, which contribute to superior electrical properties. Furthermore, it is possible to grow graphene on 3D templates using CVD and obtain a 3D graphene specimen by etching the initial metallic template.^12^

In this study, we propose a self‐sensing ionic soft actuator utilizing 3D graphene woven mesh as a signal collector. This graphene mesh electrode is composed of a monolithic network of hollow graphene tubes assembled in a mesh‐type architecture. The high electrical conductivity of graphene, along with its chemical stability, facilitates a neat and stable data acquisition during the deformation of the actuator. In addition, the wavy pattern of graphene tubes in the mesh‐type structures renders this electrode flexible and stretchable, which are crucial requirements for implementation in ionic soft actuators. The proposed graphene mesh is a plane weaved mesh that contains multiple arrays of large holes. While collecting data, the graphene mesh electrode does not impede ion motion, such that ions can freely migrate under the imposed electric field. Not only is this measurement technique beneficial in estimating the mechanical deformation of soft ionic actuators, but it could also be employed for measuring the ionic conductivity of polymer membranes and for real‐time monitoring of ion migration within ionically conductive polymers.

## 2 Results and Discussion

### 2.1 Synthesis and Characterization

**Scheme 1:**
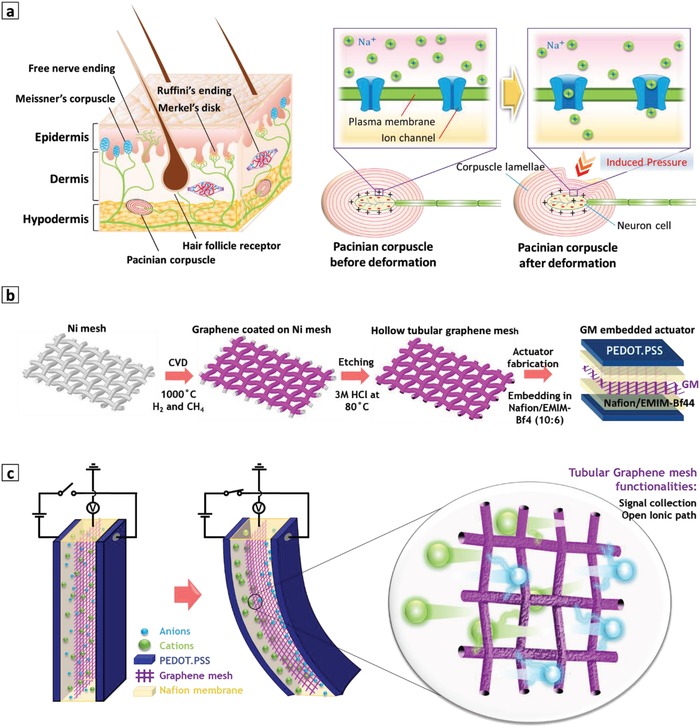
Schematic diagram of the sensing mechanism and fabrication steps of the self‐sensing actuator. a) Various mechanoreceptor in human skin and sensing mechanism of Pacinian corpuscle. b) Synthesis of graphene mesh electrode by growth of graphene on surface of Ni mesh using CVD, etching Ni in HCl solution, and embedding of mesh inside electrolyte solution. c) Actuation and sensing mechanism of self‐sensing actuator using embedded graphene mesh electrode. When ions move due to electric field, graphene mesh interacts with them and collects information about ions' migration.

The sensing mechanism of the proposed self‐sensing actuator is inspired by the reception function of human mechanoreceptors. As shown in **Scheme** 1a, the human skin is divided into three layers: epidermis, dermis, and hypodermis. In the dermis layer there exists various types of mechanoreceptor, whose operation is based on ions' movement. For a better understanding of reception mechanism, the Pacinian corpuscle could be considered. The Pacinian corpuscle is composed of onion‐type lamellae encapsulating a sensory neuron cell. The medium of the neuron cell is separated from the medium of lamellae by the plasma membrane of the cell, which comprises a network of ion channels. Initially, the ion channels are closed and the concentration of sodium ions inside the lamellae (outside the neuron cell) is higher than the concentration inside the neuron cell. When the skin is pressed by an external stimulus, the induced pressure deforms the lamellae of the corpuscle. The generated pressure inside the lamellae opens the ion channels, causing the leakage of sodium ions (Na^+^) into the neuron cell. The presence of extra Na^+^ ions alternates the potential of the neuron cell and provides the action potential for firing the neuron (Scheme 1a).

Inspired by this mechanism, we aimed at measuring the potential change inside the electrolyte membrane to trace the movement of ions during actuation. Toward this purpose, we synthesized a graphene mesh by CVD method and embedded it inside the electrolyte membrane to collect the potential data. A nickel (Ni) woven mesh was selected as a template for growing the graphene layer. The CVD method is based on catalytic growth of graphene; among a variety of transition metals, Ni has been shown to be a promising substrate for growing multilayer graphene because it allows for growth of large‐scale crack‐free graphene.^13^ Furthermore, Ni mesh is commercially available in various dimensions and patterns, which makes it an ideal choice for our applications considering time and cost efficiency. As depicted in Scheme 1b, the Ni substrate was heated to 1000 °C in ambient pressure and flushed with hydrogen and methane (100:80 s.c.c.m) as reaction gases. When the reaction was completed and carbon atoms were well dissolved in the Ni substrate, the samples were rapidly cooled to room temperature. In this step, carbon atoms were defused out of Ni and precipitated on the surface to form a crystalline multilayer graphene film. Then, a pristine free‐standing graphene mesh was obtained by etching the Ni substrate in hot 3 m hydrochloric acid (HCl) solution. Through layer‐by‐layer casting, the as‐prepared graphene mesh was later embedded inside a Nafion/1‐Ethyl‐3‐methylimidazolium tetrafluoroborate (EMIM‐Bf4) membrane. Using drop casting, the graphene‐embedded membrane was then sandwiched between two poly(3,4‐ethylenedioxythiophene) polystyrene sulfonate (PEDOT:PSS) layers as actuator electrodes. More details about material synthesis and fabrication steps can be found in the Experimental Section and Supporting Information. In the aforementioned layout, the embedded graphene mesh acts as a sensing electrode that apprehends ion movements within the polymer electrolyte membrane, without hindering the ions' migration. Indeed, large holes (≈160 µm) in the graphene mesh provide a path for ion migration, such that ions can freely move in response to the applied electric field (Scheme 1c).

**Figure 1:**
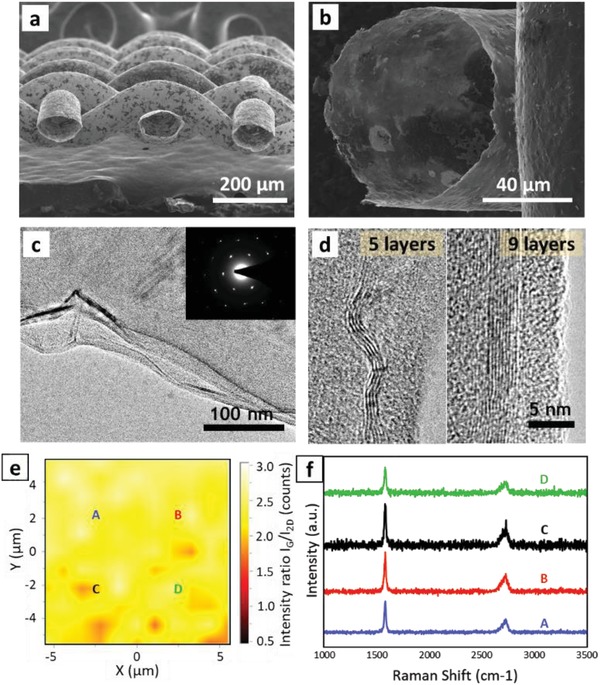
Characterization of prepared graphene mesh. a) SEM image of pristine graphene mesh at low magnitude. b) SEM image of pristine graphene mesh at high magnitude. c) TEM image of graphene sample at low magnitude. The inset shows the electron diffraction pattern of the prepared graphene sample. d) High magnitude TEM image from cross‐sectional view of graphene sample displaying number of layers. e) Mapping of peak intensity ratio, *I_G_*/*I*_2D_, in Raman Spectra captured from 10 µm  ×  10 µm area on prepared graphene mesh. f) Raman spectra corresponding to points A, B, C, and D on mapping plot.

The as‐prepared graphene mesh was first characterized by scanning electron microscopy (SEM) and transmission electron microscopy (TEM). SEM images in **Figure** 1a,b confirm that a monolithic 3D structure of graphene sheets was grown employing the CVD method. The hollow graphene warps were perfectly weaved with each other and formed a plane woven pattern. The thickness of the grown graphene was sufficient to maintain the round shape of the initial Ni wire mesh, even after etching of the Ni skeleton, as shown in Figure 1b. All graphene sheets in the lattices were thoroughly bonded to the adjacent sheets, such that no cracks or defects were observed in the SEM images of the whole surface. TEM images reveal a flat configuration of graphene sheets, as shown in Figure 1c. The electron diffraction pattern of the sample clearly shows distinguishable white spots with typical sixfold symmetry, implying the crystalline nature of the prepared graphene sample. High‐resolution TEM images of certain folded parts reveal the formation of multilayer graphene. Figure 1d identifies 5–9 graphene layers in cross‐sectional view.

To further examine the uniformity of the synthesized graphene, Raman spectra were captured from a 10 µm × 10 µm area of the sample. Figure 1e displays the mapping of the peak intensity ratio (*I_G_*/*I*_2D_) in Raman spectra. Based on the literature, the intensity of the G and 2D peaks of graphene can be used for determining the number of layers.^14^ By changing graphene from mono layer to multilayer, the intensity ratio of the G peak (at around 1580 cm^−1^) to that of the 2D peak (at around 2750 cm^−1^) increases. As illustrated in Figure 1e, the *I_G_*/*I*_2D_ ratio does not notably vary through the surface, signifying that the thickness of graphene remains similar on the whole surface. These mapping data indicate uniform graphene growth through CVD, which results in enhanced electro‐mechanical properties of the sample. Figure 1f displays the four Raman spectra corresponding to points A, B, C, and D in Figure 1e. An interesting point, that is supported by this figure, is the absence of the D peak (between 1270 and 1450 cm^−1^) at all four points. Since a D peak in Raman spectra is an indication of defects in graphene sheets, the absence of this peak at all points reaffirms the growth of high quality graphene.^14^

**Figure 2:**
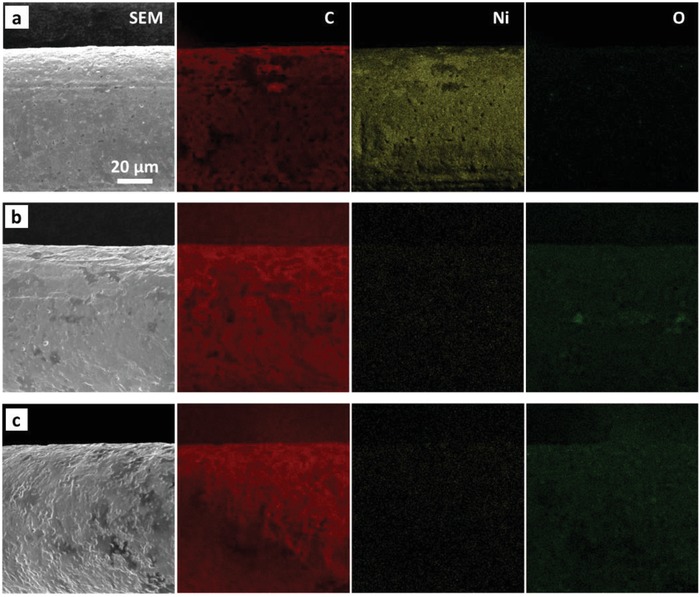
Elemental mapping of graphene mesh samples: a) Graphene grown by CVD on top of Ni mesh. b) Pristine graphene mesh after etching Ni substrate. c) Graphene mesh after being subjected to 1000 cycles of cyclic voltammetry in 0.5 m EMIM‐Bf4/Acetonitrile electrolyte.

In order to investigate the chemical stability of the as‐prepared graphene mesh, elemental mapping was carried out using energy dispersive X‐ray spectroscopy (EDS) on three samples: graphene coated Ni mesh right after CVD, pristine graphene mesh after etching with Ni, and graphene mesh after being subjected to 1000 cycles of cyclic voltammetry (CV). The results of these analyses are presented in **Figure** 2. The elemental mapping of the sample right after graphene growth shows a dense distribution of Ni component along with carbon because the Ni substrate beneath the graphene layers was not yet removed (Figure 2a). However, after executing the etching process in hot HCl solution, the Ni substrate was completely dissolved. As shown in Figure S3, Supporting Information, no peak corresponding to Ni atoms was observed in the spectrum of this sample. The obtained atomic percentage of Ni by this analysis was 0.03% which confirms that not only Ni was fully etched but also it was perfectly washed out by deionized (DI) water such that dissolved Ni ions did not remain on the mesh. Thus, no condensed mass of Ni component was further observed in the elemental mapping, as shown in Figure 2b. This figure also reveals that the concentration of the oxygen component slightly increased, which was expected because the Ni substrate was etched in hot 3 m HCl solution (a highly oxidative medium). As explained earlier, the sample was then subjected to 1000 CV cycles in EMIM‐Bf4/Acetonitrile solution (0.5 m) to inspect the chemical stability of the graphene mesh in the electrochemical reactions that might be involved in the actuation process. The elemental mapping of this sample did not show a meaningful change in the oxygen content when compared with the sample before CV (Figure 2c). This confirms that graphene is highly stable in EMIM‐Bf4 electrolyte and has been properly selected for working in such medium.

**Figure 3:**
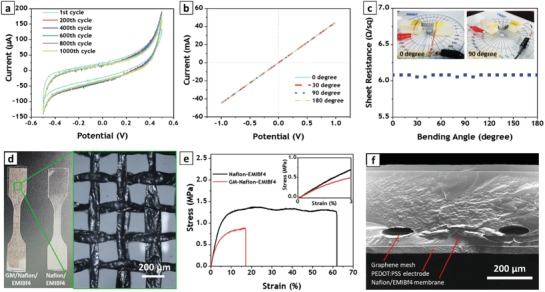
Electromechanical and electrochemical characterization of prepared graphene mesh electrode. a) Cyclic voltammetry test of graphene mesh in 0.5 m EMIM‐Bf4/Acetonitrile electrolyte for 1000 cycles. The scan rate was 100 mV s^−1^ and potential window was (−0.5 to 0.5 V). b) Current–voltage characteristic of as‐prepared graphene mesh electrode at different bending angles. c) Sheet resistance of graphene mesh electrode at different bending angles. Inset is optical image of measurement setup at 0° and 90° bending angles. d) Optical photo of tensile test samples. The inset is a magnified image of the graphene mesh in Nafion/EMIM‐Bf4 membrane captured by optical microscope. e) Stress/strain graph of electrolyte membrane with (red) and without (black) graphene mesh. The inset shows stress/strain graph for initial 3% elongation. f) SEM image captured from cross‐sectional view of fabricated self‐sensing actuator.

**Figure** 3a selectively shows six cycles of the CV test. Data presented in this graph point at a stable CV with negligible change in current density during 1000 cycles. These results demonstrate the capability of the graphene electrode for working in processes that involve multiple charging–discharging cycles, like those of ionic actuators. Given the necessity of electrode bending during actuation, the electro‐mechanical properties of the graphene electrode are critically important. To examine the electrical properties of the mesh during mechanical deformation, we employed two‐probe resistance measurement. Using silver paste, two copper foil electrodes were attached to both ends of the graphene mesh and the whole electrode system was mounted on two polymer cubes. While the polymer cube at one end was held fixed, the one at the other end was relocated to bend the graphene mesh into different angles (see Figure S4, Supporting Information). As shown in Figure 3b, the current–voltage characteristics of the graphene electrode were measured at various bending angles. The graph in Figure 3b highlights two important features of the as‐prepared electrode. First, the current–voltage curve of the sample is perfectly linear for all bending angles, emphasizing the inherent metallic conductivity of the graphene electrode. Second, the slope of the current–voltage curve remains intact at all bending angles, causing the curves to completely overlap. The calculated electrical resistances for different bending angles are presented in Figure 3c. The as‐prepared graphene mesh demonstrates a negligible change of electrical resistance during bending. This electrode showed very low electrical resistance of 6.25 Ω Sq^−1^ even at 180° bending deformation. This resistance value is prominent when compared to other previously reported graphene electrodes, pointing at a high electrical conductivity of the proposed electrode.^11^, ^15^

Thereafter, employing layer by layer casting of the Nafion/EMIM‐Bf4 (5:3 wt) solution and drying, the graphene mesh electrodes were embedded inside electrolyte membrane. To study the effect of the graphene mesh on the mechanical properties of the electrolyte membrane, a tensile test was conducted. As illustrated in Figure 3d, two specimens were prepared: a pristine electrolyte membrane and an electrolyte membrane with embedded graphene mesh. The optical microscopy of the electrolyte membrane with embedded graphene mesh shows that the mesh was neither torn nor destroyed during casting and drying procedure, and no trapped bubbles were observed in the sample (see the inset of Figure 3d). The tensile test revealed that the strength of the membrane notably decreased due to the embedding of the graphene mesh (Figure 3e). A possible cause can be the presence of void areas inside the hollow graphene tubes, which reduce the mechanical strength of the whole membrane. However, the tensile strength does not play a role in our target application, since the actuator operating strain is much lower than the tensile strength of the prepared composite membrane.[qv: 2a,b,3d,16] The maximum bending strain for the proposed self‐sensing actuator does not exceed 1%. The inset of Figure 3e shows the strain‐stress diagram of both samples within the first 3% strain window. We note that the mechanical stiffness of the membrane with embedded graphene mesh (GM/Nafion/EMIM‐Bf4) is less than that of the pure electrolyte membrane (Nafion/EMIM‐Bf4), which suggests that adding the graphene mesh reduces the stiffness of the membrane and makes it much softer and easier to bend.

Following the characterization of the proposed composite membrane, the actuator was fabricated by drop‐casting of PEDOT:PSS electrodes on both sides of the membrane. Details about actuator fabrication steps and wiring of the electrodes are provided in the Experimental Section, as well as Supporting Information. The SEM image taken from the cross‐sectional view of the actuator is displayed in Figure 3f, which shows that the graphene mesh was properly embedded inside the electrolyte membrane without any contact with the actuator electrodes. As clarified by the image, the distance of the graphene mesh from the actuator electrodes needs to be unequal; this will be explained later. The actuator electrodes (PEDOT:PSS) were also well coated on the electrolyte membrane, having a uniform thickness along the surface and firm contact with the membrane.

### 2.2 Sensing‐Actuation Measurements

**Figure 4:**
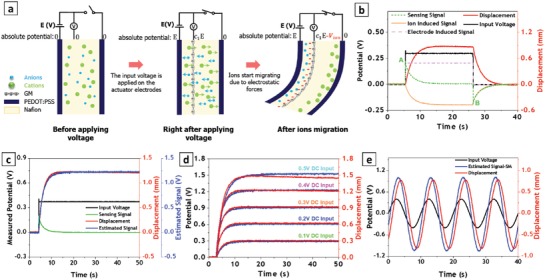
Sensing‐actuation measurement of fabricated self‐sensing actuator: a) Schematic diagram of sensing mechanism. b) Plot of acquired signals and components of sensing signal. c) Real and estimated displacement signals obtained by static model for self‐sensing actuator stimulated by 0.4 V DC voltage. d) Real and estimated displacement signals obtained by static model for self‐sensing actuator stimulated by different DC voltages. e) Real and estimated displacement signals obtained by static model for self‐sensing actuator stimulated by 0.4 V AC voltage.

After preparation, the actuator was tested for sensing‐actuation performance by applying an input electric potential across its electrodes. While performing the actuation test, we measured the absolute potential at the embedded graphene mesh electrode and the tip displacement of the actuator by a laser sensor to provide a reference value. To better illustrate the sensing mechanism, a schematic diagram of the proposed self‐sensing actuator is provided in **Figure** 4a. Before applying the electrical stimulation, ions are uniformly distributed through the electrolyte membrane and no potential develops at any of electrodes.

By applying a constant input potential (*V*_in_), one of the actuator electrodes experiences an absolute potential of zero (anode), while the other electrode experiences an absolute potential of *V*_in_ (cathode). This potential difference generates an electric field through the membrane, which induces an absolute potential on the graphene mesh electrode, located between the actuator electrodes. Such an induced potential is a fraction of the input voltage, and it depends on the location of the mesh with respect the anode. At the same time, the electric field inside the electrolyte membrane causes the differential migration of the cations and anions, which, in turn, generates a uneven charge distribution along the thickness of the membrane that manifests into a varying electrical potential on the graphene mesh electrode (asymmetrically along the thickness of the membrane). As sketched in Figure 4a, the graphene mesh electrode will be closer to the anions pile‐up, which would cause a negative potential on the mesh. We hypothesize that the measured signal on the graphene mesh is the combination of two electrical potentials, one that is elicited by the external electrodes and one that is associated with the ion redistribution.

Experimental results seem to confirm this phenomenological explanation. As shown in Figure 4b, upon applying a DC voltage, the sensing signal suddenly jumps to a positive value between zero and the input potential value (point A on the green dotted‐line), which is due to initial electric field. As ions migrate through the membrane thickness, the sensing signal gradually decreases due to the pile‐up of the anions close to the mesh electrode. Upon removing the input voltage, the potential induced by the actuator electrodes vanishes, while the negative potential associated with the ions' redistribution remains. This is because the ions cannot immediately resume their original configuration as the voltage stimulus is set to zero. As a result, the sensing signal abruptly drops to a negative value (point B on the green dotted‐line), before gradually going back to zero as the ions complete their migration to the initial electroneutral configuration. An alternative explanation based on a circuit model for binary electrolytes^17^ is presented in the Supporting Information.

Consequently, the sensing signal can be decomposed into its contributions: the electrodes‐induced potential (*V*_e_) and ions‐induced potential (*V*_ion_). These signals are also shown in Figure 4b by pink dashed‐line and orange solid‐line, respectively. We write the sensing signal as follows:

(1) $$V_{\mathrm{sense}}=V_{e}\;+V_{\mathrm{ion}}$$

Since the bending deformation of ionic actuators is controlled by osmotic and electrostatic forces, which are both triggered by the migration of unequally sized cations and anions within the membrane, one should expect a robust correlation between the bending displacement of the actuator and the ions‐induced voltage, *V*_ion_. The latter, in fact, contains salient information regarding the distribution of ions through thickness of the membrane, which ultimately determine osmotic pressure and electrostatic forces.^18^

A closer look at Figure 4b seems to confirm this claim. As one can see in Figure 4b, the ions‐induced signal is very similar to the real displacement measured by an external laser sensor (compare orange and red lines.). The ions‐induced potential can be easily extracted from the sensing signal by considering that the electrodes‐induced voltage is proportional to the input voltage (*V*_in_):

(2) $$V_{e}=C_{1}\;V_{\mathrm{in}}$$

By substituting Equation (2) into Equation (1), the ions‐induced potential can be obtained as follows:

(3) $$V_{\mathrm{ion}}\;=\;V_{\mathrm{sense}}\;-\;C_{1}V_{\mathrm{in}}$$

By multiplying the ions‐induced signal by another constant, one can estimate the actuator displacement.

(4) $$\delta_{SM}=C_{2}\;\left(V_{\mathrm{sense}}-C_{1}V_{\mathrm{in}}\right)$$

where δ_*SM*_ is the estimated tip displacement of the self‐sensing actuator. Beyond supporting evidence by the circuit model, the proposed explanation can be grounded in the distributed model of Nemat‐Nasser and Li,^19^ as shown in the Supporting Information.

Employing linear regression, the coefficients *C*_1_ and *C*_2_ can be estimated from one set of signals acquired from a specific input stimulus and then utilized to explore other inputs. Figure 4c demonstrates the estimated displacement for an input potential of 0.4 V using the presented model. A precise match can be clearly seen between the estimated signal and the real displacement data measured via the external laser sensor. The estimation error for this case is only 0.55%. The calculated coefficients were used to estimate the displacement of the self‐sensing actuator at other input voltages. As displayed in Figure 4d, a fair match was achieved for a variety of DC inputs using the same coefficients. Even for 0.1 V stimulation, which generates very low displacement (300 µm), the final estimated displacement was very accurate (around 96%).

While the proposed model works adequately for DC inputs, it shows sluggish performance for dynamic inputs such as AC voltage. As shown in Figure 4e, the estimated displacement for sinusoidal input with peak voltage of 0.4 V shows a phase delay with respect to the real displacement. This issue was also observed in earlier theoretical studies.^19^ Therefore, we refer to the proposed mode as the Static Model (δ_*SM*_) and only use it for DC inputs. To adjust the model for dynamic inputs, a phase shifting term could be added, by considering a differential form of the static model (see Supporting Information)

(5) $$\delta_{DM}\;=\;\delta_{SM}\;\cos\varphi \;+\;\frac{1}{\omega }\delta \prime_{SM}^{}\sin\varphi$$

where δ_*DM*_ and δ_*SM*_ are the tip displacements of the actuator estimated through the dynamic and static Models, respectively. In this equation, ω is the frequency of the harmonic input potential, while φ is the phase delay of the static model. As in the static model, the coefficients *C*_1_ and *C*_2_ along with the phase shift φ in the dynamic model can be estimated from regression for a specific input potential and then used for other inputs.

**Figure 5:**
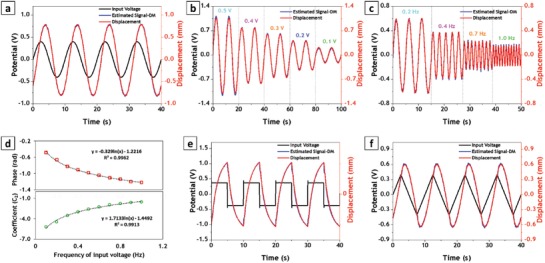
Sensing‐actuating measurement of fabricated self‐sensing actuator for alternating stimulations: a) Real and estimated displacement signals obtained by dynamic model for 0.4 V AC stimulation. b) Real and estimated displacement signals for different amplitudes of AC stimulation. c) Real and estimated displacement signals for different frequencies of AC stimulation. d) Variation of estimated phase shift and amplitude coefficient (*C*_2_) for different frequencies of AC stimulation. e) Real and estimated displacement signals for 4 V square wave stimulation. f) Real and estimated displacement signals for 4 V triangle wave stimulation.

As shown in **Figure** 5a, for a harmonic input with peak voltage of 0.4 V, the dynamic model produced a close match between the estimated signal and real displacement. The calculated coefficients were then utilized to estimate the response of the actuator to other amplitudes of harmonic input potentials. As one can see in Figure 5b, using the acquired sensing signal and estimated coefficients, the dynamic model successfully predicts the response of the actuator to various AC inputs with different amplitudes. Even at the very low driving voltage of 0.1 V, this sensing system can predict the bending deformation accurately, pointing at the strong sensitivity of the proposed self‐sensing actuator (see also Figure S14, Supporting Information).

The other influential parameter affecting the estimation model is the frequency of the input voltage. Figure 5c presents the displacement estimated by the dynamic model for different frequencies of AC input potentials. Although a good match is achieved using this model, the coefficient *C*_2_ and phase shift φ needed to be modified for each case, implying the frequency‐dependency of these parameters. As illustrated in Figure 5d, the calculated parameters *C*_2_ and φ are described by logarithmic functions with respect to the frequency of the input stimulation. This could be expected due to interplay of damping and inertia effects in the actuator, which is modulated by the excitation frequency.

In addition to sine wave inputs, the as‐prepared self‐sensing actuator was also tested for square wave and triangle wave inputs (Figure 5e,f). Similar to the sine wave profile, in these other cases the coefficient was calculated from one set of signals acquired for each input profile (square or triangle) and then utilized to predict the response for other inputs with the same profile. In both cases, the estimated signal properly predicted the measured displacement of the actuator, suggesting the potential use of the proposed device for in a wide range of stimulation conditions (see Figures S15 and S16, Supporting Information).

In order to inspect the life‐time stability of the proposed self‐sensing actuator, the samples were again tested after 2 months to check if their performance is degraded or not. However, as presented in Figure S17, Supporting Information, no meaningful change was observed neither in output displacement of the actuator nor in sensing signal. After 2 months, the sensing signal was still very neat and clean without any noise or disturbance.

## 3 Conclusion

Inspired by mechanoreceptors in the human body, we developed an innovative self‐sensing ionic soft actuator using graphene woven mesh electrode. The proposed 3D graphene electrode showed outstanding multi‐functional attributes, such as flexibility, high electrical conductivity, and ion permeability. Embedding a high‐quality graphene mesh inside the electrolyte membrane could afford a novel self‐sensing ionic actuator that could precisely measure the displacement during actuation. In the suggested bio‐inspired sensing mechanism, the sensing signals were generated by mobile ions inside an electrolyte membrane, similar to various types of mechanoreceptors in the human body. The signals, collected by graphene mesh, contained the information regarding the distribution of ions within the membrane and was properly correlated with the bending deflection to estimate the tip displacement of the actuator. Using the sensing data, semi‐empirical models were proposed to estimate the tip displacement for both static and dynamic electrical stimulations. These static and dynamic models properly predicted the tip displacement of the actuator at a variety of input potentials.

The obtained results offer compelling evidence in favor of this newly designed self‐sensing actuator. Not only can the proposed graphene mesh electrode and sensing mechanism be used for new types of self‐sensing actuators, but also for many other applications, such as measuring ionic conductivity of polymer membranes or monitoring ion movement inside solid‐liquid electrolytes in real‐time. Further investigation of this approach could pave the way for rigorous electrochemical studies of electrolytic membranes that could improve our understanding of ions' interactions.

## 4 Experimental Section

*Graphene Mesh Synthesis*: A plane woven Ni mesh with a pore size of 155 µm was selected as a template for graphene growth. Ni mesh substrates were cut into 1 cm × 4 cm samples and washed with ethanol. The substrates were lunched in a long quartz tube (1.5 m) and heated to 1000 °C in ambient pressure and Ar atmosphere (1000 s.c.c.m.). After reaching the reaction temperature, the samples were kept in this condition to remove the oxides and other impurities from the surface. In order to initiate the growth, reaction gases (H_2_, 100 s.c.c.m. and CH_4_, 80 s.c.c.m.) were flowed into the tube while decreasing Ar flow rate to 400 s.c.c.m. After 10 min, the H_2_ and CH_4_ flows were stopped and the furnace was removed from the samples to swiftly cool them to room temperature. The Ni substrate was etched from graphene coated samples using 3 m HCl solution while keeping samples in the oven (80 °C) for 24 h. Afterward, floating graphene samples were washed with DI water three times to remove the acid and then dried by freeze‐drying technique at −50 °C and 14 Pa pressure.

*Fabrication of Self‐Sensing Actuator*: First, Cu foil was attached to one end of graphene mesh using silver paste. Then, the electrolyte solution was prepared by dissolving 3 g Nafion and 1.8 g EMIM‐Bf4 in 30 mL Dimethylacetamide (DMAc). To achieve a well‐dissolved solution, the mixture was stirred in an oil bath (80 °C) for 24 h. Then, 2 mL of the electrolyte solution was casted in a circular glass Petri dish (diameter: 55 mm) and dried in the oven to remove the DMAc. Afterward, the as‐prepared graphene electrode was placed on the dried membrane and 0.5 mL of the electrolyte solution was drop‐casted on the graphene mesh and dried to fix the mesh in that position. Another 4 mL of electrolyte solution was casted on the dried membrane to fully embed the graphene mesh electrode inside the membrane. After obtaining the membrane with embedded graphene mesh, it was sandwiched between actuator electrodes by drop‐casting DMSO/PEDOT:PSS (5 vol%) on both sides of the membrane. Finally, the actuators were cut into favorable sizes and the sensing‐actuating measurements proceeded.

*Material Characterization*: Micro‐scale surface morphology of the graphene mesh and cross‐sectional view of the actuator were obtained using a Field Emission Scanning Electron Microscope (Magellan400) made by the FEI Company. This microscope was equipped with an Energy‐dispersive X‐ray spectroscopy system that was used for elemental mapping of the samples. Nano‐scale surface morphology and cross‐sectional views of graphene sheets were obtained using a Field‐emission transmission electron microscope (300 kV Tecnai G2 F30 S‐Twin) made by the FEI Company. High‐resolution images of the graphene mesh embedded in the electrolyte membrane were captured using an optical microscope (ECLIPSE LV100D‐U) made by the Nikon Company. Raman spectra analysis was carried out using a High‐Resolution Raman Device (LabRAM HR Evolution Visible‐NIR) made by HORIBA. Mechanical properties of electrolyte membrane were determined using a Universal/Tensile Test Machine (AGS‐X) made by SHIMADZU Corporation. Two‐probe resistance measurement of the graphene mesh was executed on a Keithley Multimeter (DMM7510).

*Actuation Measurements*: To perform the sensing‐actuating measurements, the as‐prepared actuator was clamped at one end using two electrodes that were connected to the power supply in order to transfer the driving input voltages to the actuator electrodes. The sensing signal was collected from the graphene mesh electrode at the same end. The tip displacement of the actuator was recorded at the free end using a High‐Accuracy Laser Displacement Sensor (LK‐H080) produced by KEYENCE Corporation. All signals were simultaneously acquired using a Data Acquisition System (NI‐PXI 1042Q, NI‐PXI 6252 DAQ board) made by National Instruments.

## Conflict of Interest

The authors declare no conflict of interest.

## Supporting information

Supplementary
