# Supplementary material for: Graphene Mesh for Self‐Sensing Ionic Soft Actuator Inspired from Mechanoreceptors in Human Body
Source: Adv Sci (Weinh). 2019 Oct 11;6(23):1901711. doi: 10.1002/advs.201901711 (PMC6891913; doi:10.1002/advs.201901711)
Supplement: Supplementary file 1 — Supplementary [file ADVS-6-1901711-s001.pdf]

Copyright WILEY-VCH Verlag GmbH & Co. KGaA, 69469 Weinheim, Germany, 2019.

ADVANCED SCIENCE
Open Access

Supporting Information

for *Adv. Sci.,* DOI: 10.1002/advs.201901711

Graphene Mesh for Self-Sensing Ionic Soft Actuator Inspired from Mechanoreceptors in Human Body

*Rassoul Tabassian, Van Hiep Nguyen, Sima Umrao, Manmatha Mahato, Jaehwan Kim, Maurizio Porfiri, and Il-Kwon Oh**

Supporting Information

Graphene Mesh for Self-Sensing Ionic Soft Actuator Inspired from Mechanoreceptors in Human Body

Rassoul Tabassian[1], *Van Hiep Nguyen*[1], *Sima Umrao*[1], *Manmatha Mahato*[1], *Jaehwan Kim*[1], *Maurizio Porfiri*[2,3] and *Il-Kwon Oh*[1,*]

[1] Dr. R. Tabassian, V.H. Nguyen, Dr. S. Umrao, Dr. M. Mahato, Dr. J. Kim, Prof. I.-K. Oh
Creative Research Initiative Center for Functionally Antagonistic Nano-Engineering, Department of Mechanical Engineering, Korea Advanced Institute of Science and Technology (KAIST), 291 Daehak-ro, Yuseong-gu, Daejeon 34141, Republic of Korea

[2,3] Prof. M. Porfiri
Department of Mechanical and Aerospace Engineering, Tandon School of Engineering, New York University, 6 MetroTech Center, Brooklyn, NY 11201, USA
Department of Biomedical Engineering, Tandon School of Engineering, New York University, 6 MetroTech Center, Brooklyn, NY 11201, USA

[*] Correspondence and requests for materials should be addressed to I.-K. Oh (email: ikoh@kaist.ac.kr).

Keywords: graphene mesh; mechanoreceptor; self-sensing; sensory actuator; porous electrode

Synthesizing Graphene Mesh

Figure S1 shows the experimental steps for preparation of graphene mesh. A pure Ni mesh with size of 100 mesh/in and plane woven pattern was selected as substrate for growing graphene. The Ni mesh was cut into $10\ mm \times 40\ mm$ pieces and sonicated in ethanol for 10 min to remove impurities from the surface. Samples were then lunched into a long quartz tube with length of $150\ cm$ and diameter of $10\ cm$. For CVD growth, an electrical furnace was used that can be moved along the quartz tube. First Ar (1000 s.c.c.m.) was flowed into the tube for 10 min to make an inert atmosphere. Then, the furnace was placed on the samples and started heating them up to 1000° C while Ar was still flowing. After reaching 1000° C, the samples were kept in that condition for 10 min to remove oxides and other impurities from the surface of the Ni substrates. Then, the Ar flow rate was decreased to 400 s.c.c.m. and the

reaction gases, H_2 (100 s.c.c.m.) and CH_4 (80 s.c.c.m.), were introduced into the tube to initiate the growth. During this step, methane as a carbon source was decomposed and the carbon atoms were dissolved in the Ni substrate. After 10 min the furnace was turned off and promptly removed from the samples. Rapid cooling caused the carbon atoms to out-defuse to the surface and form a crystalline carbon layer. In the cooling step, H_2 and CH_4 were stopped and only Ar was kept flowing. To obtain a pristine graphene mesh, the Ni substrate was removed by $HCl.$ A 3M HCl solution was prepared and samples were kept in the solution for 24 hours at 80° C. After etching process, the color of solution changed to green and pristine graphene samples floated on top of the solution. The samples were then washed with DI water 3 times to remove the acid. To prevent the 3D graphene structure from collapsing during drying, a freeze-drying technique was employed. For this, the samples were first collected on glass slides. Then, the glass slides were placed on an ice substrate and frozen. If samples be frozen while they are in the water, the growth of ice crystals during freezing could damage the graphene sample (see Figure S2). After freezing, the samples were dried by sublimating the ice into gas using very low temperature and pressure (-50° C and 14 Pa). Free-standing pristine graphene mesh was achieved after 12 hours when the whole water is removed.

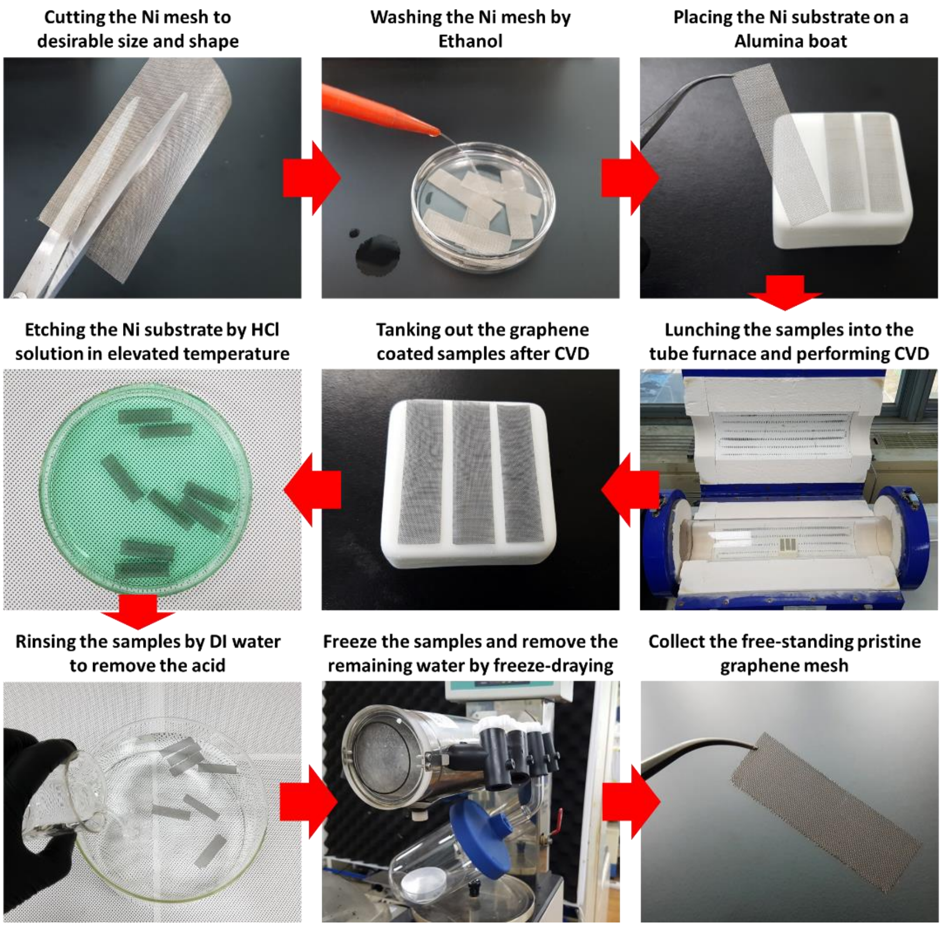

Figure S1. Experimental steps for synthesizing pristine graphene mesh.

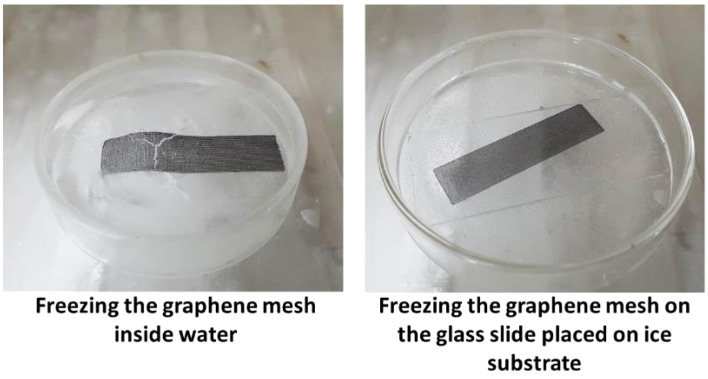

Figure S2. Formation of cracks due to growth of ice crystals during freezing process.

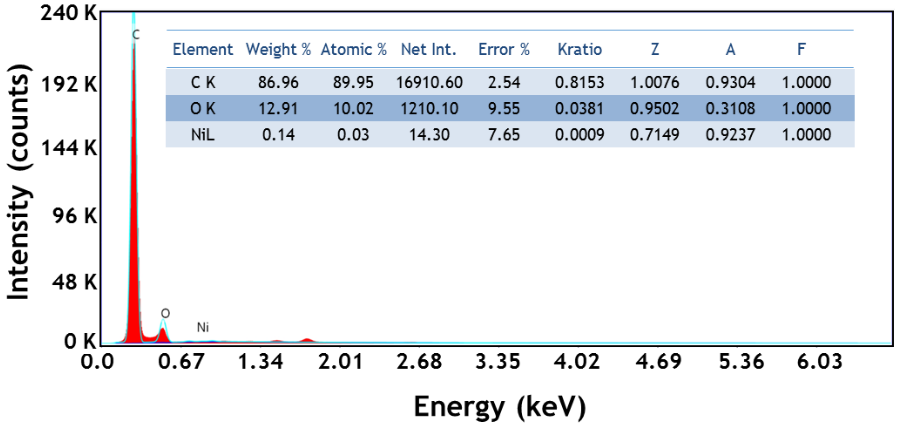

Figure S3. The energy-dispersive X-ray spectrum of the pristine graphene mesh after dissolving *Ni* substrate.

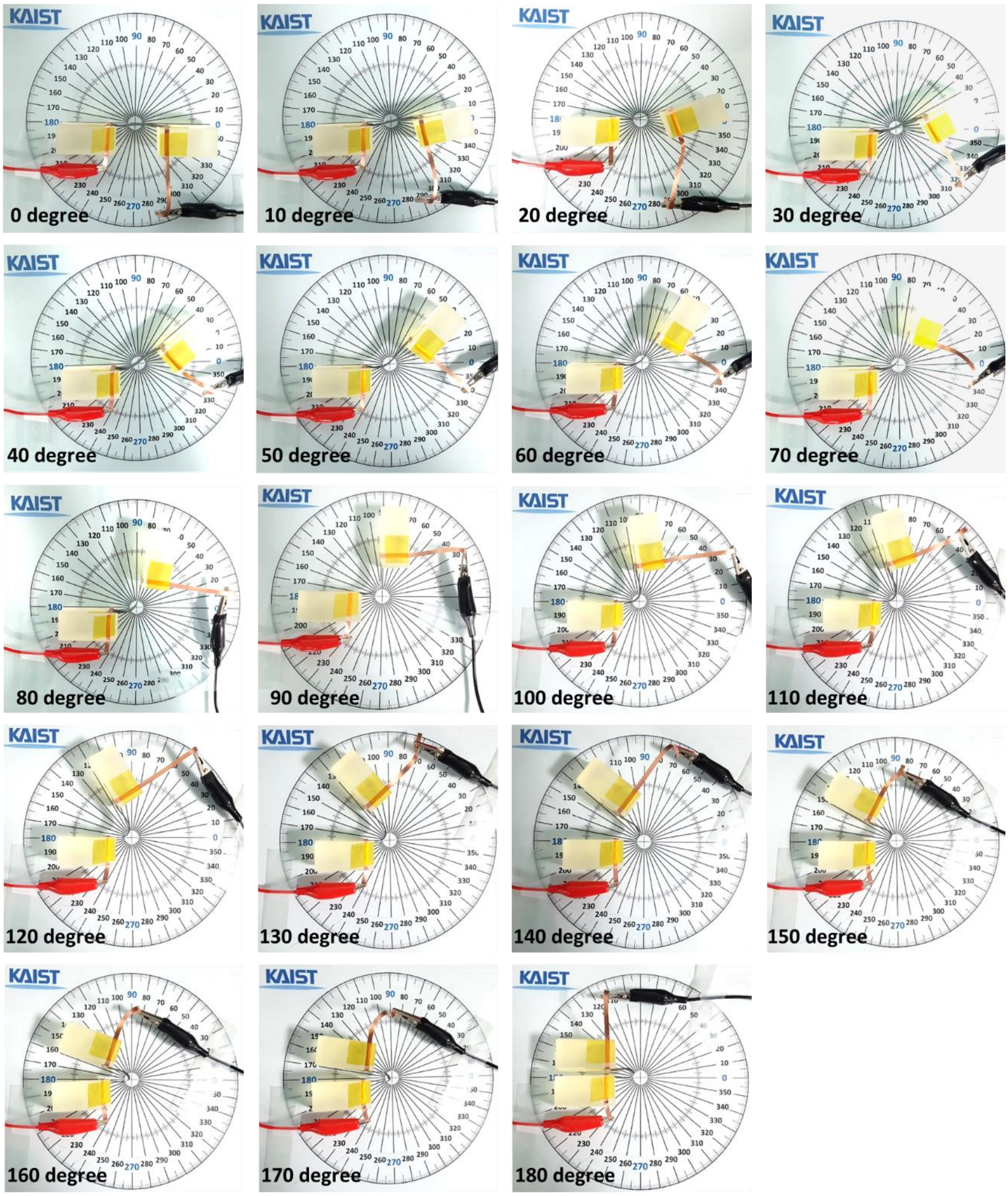

Figure S4. Measurement of Current-Voltage characteristic and electrical resistance while bending the graphene mesh into different angles (0 to 180 degrees).

Fabricating Self-Sensing Ionic Actuator

The fabrication steps of the proposed self-sensing actuator are presented in Figure S5. First, the electrolyte solution was prepared by dissolving Nafion/EMIM-Bf4 (10:6 wt) in Dimethylacetamide (DMAc). The content of DMAc was not important since it was only used as a solvent and was finally evaporated. In our case, the concentration of Nafion in DMAc was 100 mg/ml. Wiring of the graphene mesh was carried out by attaching a copper electrode. To do so, first one end of the graphene mesh was placed on a thin polyethylene film (commercial wrapping films). Then, using silver paste, a copper foil strip was attached to the graphene (Figure S5). Since the silver paste was brittle after drying, a thin layer of PDMS was drop coated on the wiring portion to encapsulate that part between polyethylene film and PDMS. This protective layer also prevented oxidation of copper inside the electrolyte solution during casting of the electrolyte at elevated temperatures (see Figure S6).

Next, 2 ml of electrolyte solution was casted in a circular glass Petri dish with the diameter of 55 mm and dried at 85° C. Afterward, the as-prepared graphene mesh electrode was placed on the dried membrane and 0.5 ml of the electrolyte solution was drop coated on the mesh and dried. This step was performed to keep the graphene electrode in fixed position with respect to the lower surface of the membrane. Another 4 ml of electrolyte solution was again casted on top of the mesh and dried in the same condition. After embedding the graphene mesh electrode inside the electrolyte, the as-prepared membrane was detached from the glass mold and then used for actuator preparation.

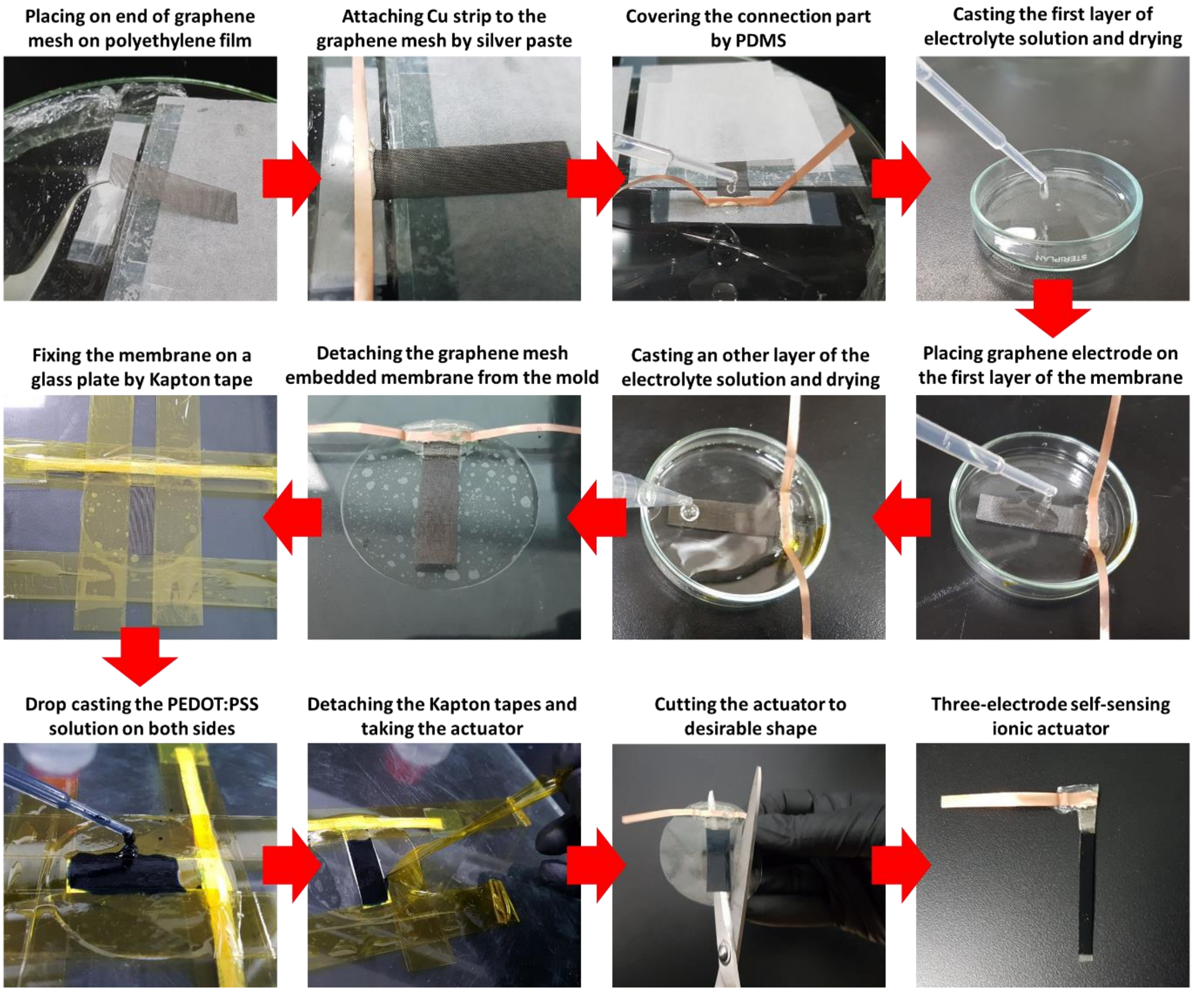

Figure S5. Fabrication steps of proposed self-sensing ionic actuator.

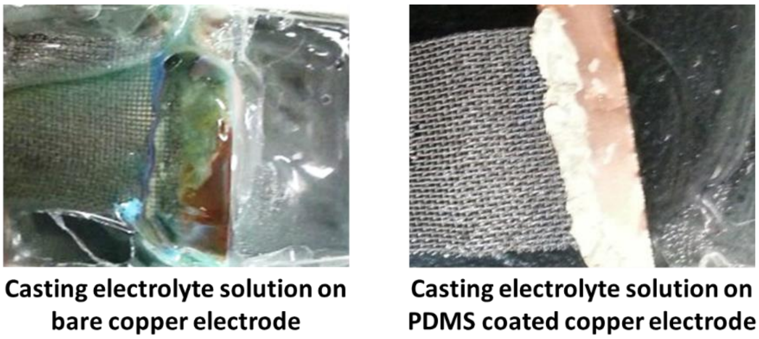

Figure S6. Oxidation of *Cu* electrode during casting process of electrolyte solution.

Before coating the actuator electrodes, the membrane was examined to find out if it is contaminated by copper oxides during casting process. For this aim, Energy-dispersive X-ray spectroscopy (EDS) and Fourier Transform Infrared spectroscopy (FTIR) were employed. As shown on Figure S7 no peak corresponding to *Cu* atoms were observed in the spectrum of the as prepared membrane. The FTIR spectroscopy were conducted for three different membranes: a) pure Nafion, b) Nafion mixed with EMIM-Bf4 (10:6 wt) without *Cu* and GM electrodes, c) the membrane of the proposed self-sensing actuator which was composed of Nafion/EMIM-Bf4 with embedded *Cu* and GM electrodes. As shown in Figure S8, no change or peak shift was observed between the spectrum of the membranes b and c. This results imply that embeding the sensing electrode inside actuator membrane doese not contaminate the electrolyte membrane neither by copper oxides nor by *Ni* ions.

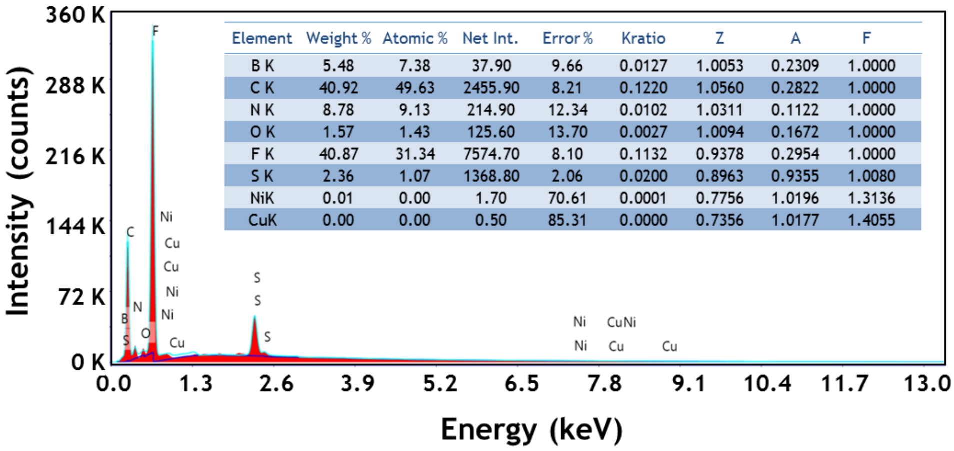

Element	Weight %	Atomic %	Net Int.	Error %	Kratio	Z	A	F
B K	5.48	7.38	37.90	9.66	0.0127	1.0053	0.2309	1.0000
C K	40.92	49.63	2455.90	8.21	0.1220	1.0560	0.2822	1.0000
N K	8.78	9.13	214.90	12.34	0.0102	1.0311	0.1122	1.0000
O K	1.57	1.43	125.60	13.70	0.0027	1.0094	0.1672	1.0000
F K	40.87	31.34	7574.70	8.10	0.1132	0.9378	0.2954	1.0000
S K	2.36	1.07	1368.80	2.06	0.0200	0.8963	0.9355	1.0080
NiK	0.01	0.00	1.70	70.61	0.0001	0.7756	1.0196	1.3136
CuK	0.00	0.00	0.50	85.31	0.0000	0.7356	1.0177	1.4055

Figure S7. The energy-dispersive X-ray spectrum of the prepared membrane for the proposed self-sensing actuator.

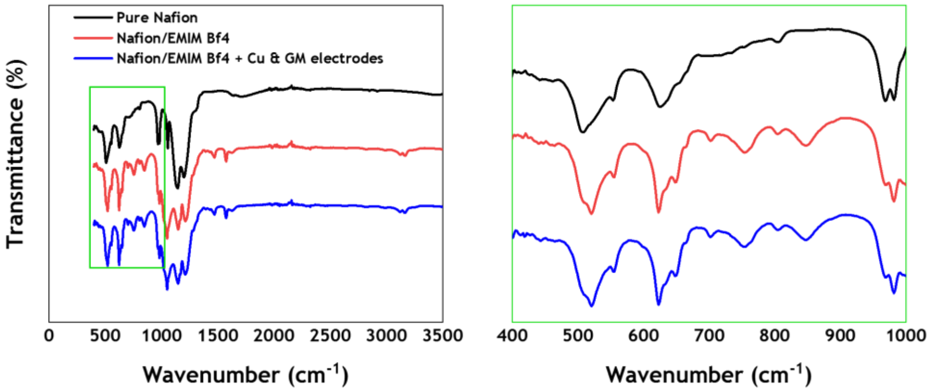

Figure S8. The FTIR spectrum of three Nafion samples: pure Nafion (black line), Nafion/EMIM-Bf4 10:6 wt (red line), and Nafion/EMIM-Bf4 with embedded *Cu* and GM electrodes (blue line).

Afterward, the electrode solution for the actuator was prepared by adding 5 vol% Dimethyl sulfide (DMSO) to PEDOT:PSS and stirring for 1 h. The edges of the graphene embedded membrane were fixed on the glass plate using Kapton tape. Then, DMSO/PEDOT:PSS solution was drop-coated on the membrane and dried in air to form a flexible electrode. Electrode coating was performed one more time on the other side of the membrane by applying the same steps. After sandwiching the membrane between the flexible electrodes, the actuator was cut into a favorable shape.

Generation of Sensing Signal

For measuring the absolute electric potential of any object, the potential difference between earth and the target object is usually measured since the earth is considered as zero potential. Accordingly, we embedded the graphene mesh into the electrolyte membrane to measure the absolute potential at a specific position inside the membrane. Regardless of ions, the actuator could be considered as a capacitor with two parallel electrodes. When a potential difference (V_{in}) is applied to the actuator electrodes, a uniform electric field is generated between the electrode whose intensity would be calculated as follows:

$$E = \frac{V_{in}}{t} \quad \text{(S1)}$$

where, t is the thickness of the actuator membrane. It is known that the potential difference between every two points inside a uniform electric field is proportional to the intensity of the electric field and the distance between two points. Accordingly, the potential difference between points M and B in Figure S9, due to the input electric field, could be calculated as follows:

$$V_{M-e} - V_B = dE \quad \text{(S2)}$$

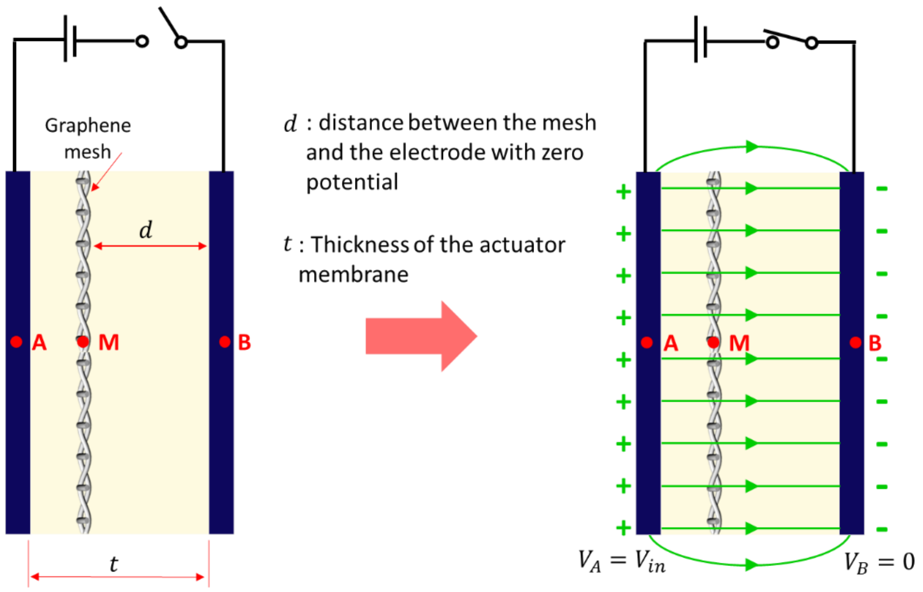

Figure S9. Generation of electric potential on the mesh due to formation of uniform electric field between parallel electrodes of the actuator.

Substituting E from Equation S1 into Equation S2 and considering $V_B = 0$, one can obtain the absolute potential at the mesh position (V_{M-e}):

$$V_{M-e} = \frac{d}{t} V_{in} \quad \text{(S3)}$$

Therefore, without considering the effect of ions, the absolute potential of the mesh should be some portion of the input voltage, the same as what was proposed by Equation 2 in the manuscript. This signal is equivalent to the purple dash line in Figure 4b (--- Electrode

induced signal). However, in ionic actuators the presence of ions also affect the internal potential of the actuator. It is known that electric potential around any type of point-charge decreases with the distance from that point-charge:

$$V = k\frac{Q}{r} \tag{S4}$$

where, k is constant and equal to $9.010^9\ \mathrm{N\,m^2/C^2}$.

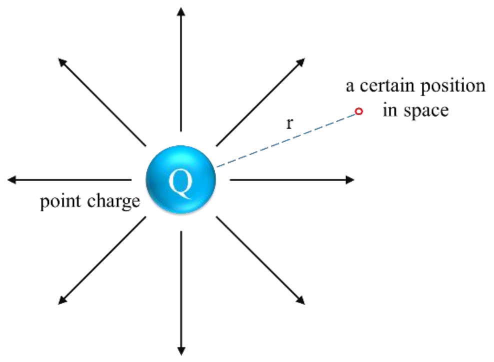

Figure S10. Generation of electric potential around a point charge in space.

Cations and anion could be simply considered as positive and negative point-charges, respectively. Therefore, the absolute potential induced by ions at the mesh position would be the superposition of the potentials that are generated by all cations and anions.

$$V_{M-ion} = k\sum_{i=1}^{n}\left(\frac{Q_{ci}}{r_{ci}} + \frac{Q_{ai}}{r_{ai}}\right) \tag{S5}$$

Where, Q_{ci} and Q_{ai} are the charge of cations and anions respectively and r_{ci} and r_{ai} are the distance of cations and anions from the mesh. The cations and anions are equally charged, therefore:

$$Q_{ai} = -Q_{ci} = Q \tag{S6}$$

$$V_{M-ion} = k\sum_{i=1}^{n}\left(\frac{Q_{ci}}{r_{ci}} + \frac{Q_{ai}}{r_{ai}}\right) \tag{S7}$$

In the neutral state, since the cations and anions are evenly distributed in the whole membrane, the potential induced by cations and anions cancels out with each other. Therefore the total induced potential by ions would be zero in the neutral state.

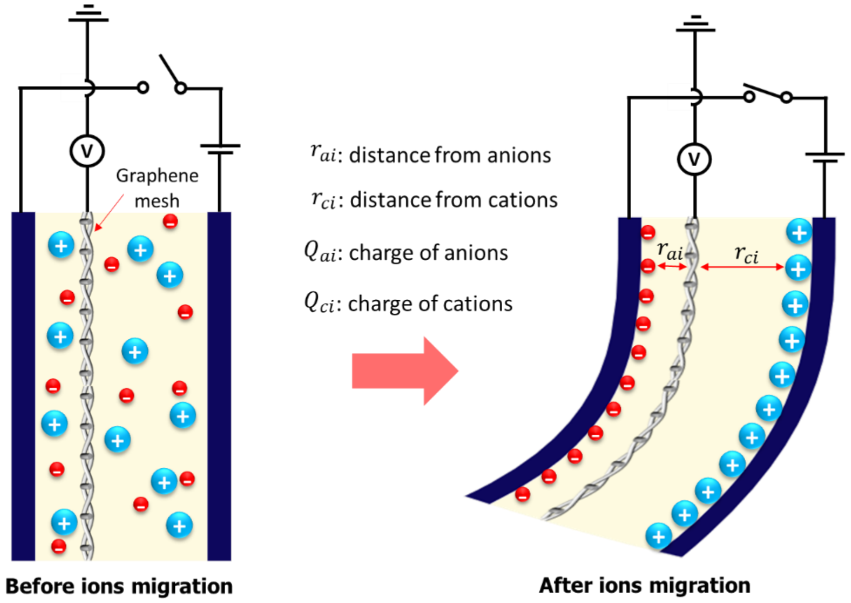

Figure S11. Generation of electric potential on the mesh due to redistribution of ions inside the actuator membrane by applying electric field.

However, the ion migration, which initiates the actuation, leads to separation of cations and anion toward opposite sides of electrodes. In this state, since the graphene mesh is closer to the anion site ($r_{ai} \ll r_{ci}$), the negative potential induced by anions is much bigger than the positive potential induced by cations. Therefore, the overall value of the ion-induced potential in Equation S7 would be a negative value. The more ions separation proceeds the more negative potential would be generated. This signal is equivalent with the orange line in Figure 4b (— Ion induced signal).

Therefore, the overall generated potential on the mesh should be combination of "Electrode-induced signal" and "Ion-induced signal" which were calculated by Equation S3 and S7 respectively.

$$V_{sense} = V_{M-e} + V_{M-ion} \quad \text{(S8)}$$

This overall sensing signal is shown in Figure 4b by green dot line (sensing signal).

Circuit model of the actuator

Within the classical framework of binary electrolytes [17], the electrical response of the self-sensing actuator can be modelled as the series connection of two *RC* impedance (**Figure S12**). In this simple voltage partitioning circuit, one of the impedances encapsulates charge dynamics in the electrolyte membrane between the anode and the mesh, and the other corresponds to charge dynamics between the cathode and the graphene mesh. The resistors are associated with the diffusion of the charges in the electrolyte membrane, while the capacitors capture the formation of electric double layers in the vicinity of the outer electrodes and the graphene mesh, as well the polarization of the membrane. In general, the values of the resistances and the capacitances are different to incorporate geometric and physical asymmetries of the self-sensing actuator. With respect to the assembly of the distance of the graphene mesh from the actuator electrodes, one should expect that $R_1<R_2$ and $C_1>C_2$, since the graphene mesh is closer to the cathode such that charges will need to migrate over a smaller portion of the membrane and the effective thickness of the capacitor will be smaller.

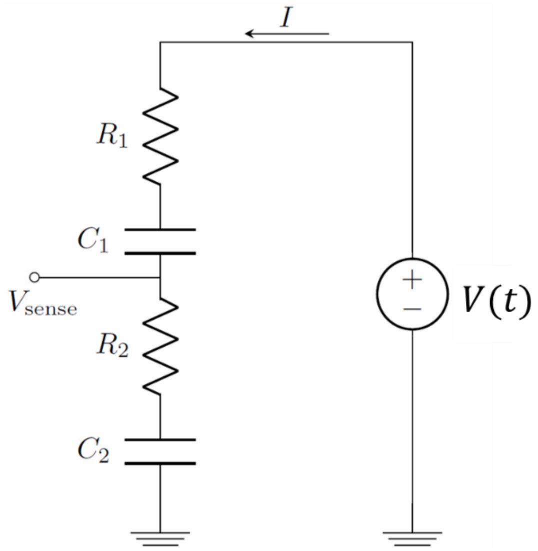

Figure S12. Schematics of the equivalent circuit for the proposed self-sensing actuator. The two RC circuits represent the two segments in which the actuator is separated by the graphene mesh electrode. The voltage sensed by the embedded electrode is denoted by V_{sense} .

Assuming that the capacitors have initially zero charge and using basic circuit theory (Kirchhoff's equation and the constitutive response for the impedances), the Laplace transform of the current $I(t)$ is:

$$L[I(t)] = \frac{L[V(t)]}{\left(R_1 + \frac{1}{sC_1}\right) + \left(R_2 + \frac{1}{sC_2}\right)} \quad \text{(S9)}$$

Where, s is the Laplace variable and $L[\ ]$ indicates Laplace transform. Hence, we can derive the following transfer function in the Laplace domain that relates the Laplace transform of the applied voltage $V(t)$ to the voltage at the graphene mesh $V_{sense}(t)$:

$$F(s) = \frac{L[V_{sense}(t)]}{L[V(t)]} = \frac{R_2\left(1 + \frac{1}{s\tau_2}\right)}{R_1\left(1 + \frac{1}{s\tau_1}\right) + R_2\left(1 + \frac{1}{s\tau_2}\right)} \quad \text{(S10)}$$

where $\tau_1 = R_1C_1$ and $\tau_2 = R_2C_2$ are the time constants of the two impedances. This transfer function can be conveniently rewritten as

$$F(s) = \alpha\frac{s - z}{s - p} \quad \text{(S11)}$$

where $\alpha = \frac{R_2}{R_1 + R_2}$ measures the contrast between the two resistances, and $z = -\frac{1}{\tau_2}$ and $p = -\frac{1}{R_1 + R_2}\left(\frac{R_1}{\tau_1} + \frac{R_2}{\tau_2}\right)$ are the (negative) zero and pole, respectively. Note that if the two impedances are equal, $\alpha = 1/2$ and $z=p$, so that $F(s) = 1/2$ and the voltage measured at the graphene mesh should closely follow the applied voltage. Including asymmetries in the self-sensing actuator causes a richer sensing response, as explained in what follows.

For the case of an applied voltage in the form of a pulse of amplitude V_0 and duration T, the Laplace transform of $V(t)$ is

$$L[V(t)] = V(s) = \frac{V_0}{s}(1 - e^{-Ts}) \tag{S12}$$

Hence, from Equation S11, we compute the Laplace transform of the voltage at the graphene mesh

$$L[V_{sense}(t)] = \alpha V_0 (1 - e^{-Ts}) \left(\frac{z/p}{s} + \frac{1 - z/p}{s - p} \right) \tag{S13}$$

By taking the inverse Laplace transform of the Equation S12, we determine the complete expression for the voltage at the graphene mesh

$$V_{sense}(t) = \begin{cases} \alpha V_0 \left[\frac{z}{p} + \left(1 - \frac{z}{p}\right) e^{pt}\right] & \text{for } 0 < t < T \\ \alpha V_0 \left(1 - \frac{z}{p}\right) \left[e^{pt} - e^{p(t-T)}\right] & \text{for } t > T \end{cases} \tag{S14}$$

For $0 < t < T$, the voltage sensed by the graphene mesh decays exponentially from a nonzero initial value, associated with the formation of the electric double layers. While the initial value of the voltage depends only on the ratio of the resistances through α, the final value is controlled by both the ratios of the resistances and of the capacitances. In the limit of T larger than the time constants of the two impedances this value depends only on the capacitances $C_1/(C_1+C_2)$. When the electrodes are shorted, at $t = T$, the voltage shows a negative spike, due to the displacement current, which is the followed by an exponential decay to zero.

Theoretical Model for Estimation of Tip Displacement

To estimate the electric field and charge distribution inside the electrolyte membrane, Poisson equation (Equation S15 and S16) and the continuity equation (Equation S17), were used respectively [19]:

$$\mathbf{E} = \frac{\mathbf{D}}{\kappa_e} = -\nabla \emptyset \tag{S15}$$

$$\nabla . \mathbf{D} = \rho = F(C^+ - C^-) \tag{S16}$$

$$\nabla.\mathbf{J} = -\frac{\partial C^+}{\partial t} + \frac{\partial C^-}{\partial t} \tag{S17}$$

Here, $\mathbf{E}$ is the electric field, $\mathbf{D}$ is the electric displacement, κ_e is the dielectric permittivity, ϕ is the electric potential, ρ is the charge density, F is Faraday's constant, C^+ and C^- are the cation and anion concentrations, and $\mathbf{J}$ is the current flux density. By neglecting the migration of the anions having smaller molecular size and using a linearized form of the Nernst-Planck equation for the constitutive response of the current flux density, Nemat-Nasser and Li obtained the following governing differential equation for the electric field along the thickness at the steady-state [19]:

$$\frac{\partial^2 E}{\partial x^2} - a^2 E = 0 \tag{S18}$$

$$a^2 = \frac{C^- F^2}{\kappa_e RT}(1 - C^- \Delta V)$$

where, R is the gas constant, T is the temperature and ΔV is the volumetric change. In this differential equation x denotes the space coordinate along the thickness direction. The standard solutions for Equation S18 is as follows:

$$E = b_1 \sinh(ax) + b_2 \cosh(ax) \tag{S19}$$

Considering $\rho = \kappa_e\, \partial E/\partial x$ and $\partial\phi(x)/\partial x = -E$, one can obtain the charge density and the electric potential by differentiating and integrating Equation S19 respectively

$$\rho = \kappa_e[c_1 a^2 \cosh(ax) + c_2 a^2 \sinh(ax)] \tag{S20}$$

$$\phi = -c_1 \cosh ax - c_2 \sinh ax + c_3 \tag{S21}$$

where $c_1 = b_1/a$ and $c_2 = b_2/a$. Throughout the modeling development, it is supposed that the origin of the spatial coordinate is located on the neutral axis of the actuator for bending deformations [20].

To determine the coefficients for Equation S21, boundary conditions need to be applied. Considering the input potential on the actuator electrodes as a boundary condition on the membrane faces, two of the unknown coefficients in the potential equation (Equation S21)

can be obtained in terms of the remaining coefficient. As shown in Figure S13 when an input potential (V_{in}) is applied to the actuator, one of the actuator electrodes experiences absolute potential of zero, while the other electrode experiences absolute potential of V_{in}:

$$\phi(-h_1) = 0 \tag{S22}$$

$$\phi(h_2) = V_{in} \tag{S23}$$

Applying these conditions to the Equation S21 yields:

$$-c_1 \cosh(ah_1) + c_2 \sinh(ah_1) + c_3 = 0 \tag{S24}$$

$$-c_1 \cosh(ah_2) - c_2 \sinh(ah_2) + c_3 = V_{in} \tag{S25}$$

Solving Equation S24 and S25 for c_2 and c_3 one can obtain:

$$c_2 = \frac{c_1[\cosh(ah_1) - \cosh(ah_2)] - V_{in}}{\sinh(ah_1) + \sinh(ah_2)} \tag{S26}$$

$$c_3 = \frac{\sinh(ah_1 + ah_2)}{\sinh(ah_1) + \sinh(ah_2)} c_1 + \frac{\sinh(ah_1)}{\sinh(ah_1) + \sinh(ah_2)} V_{in} \tag{S27}$$

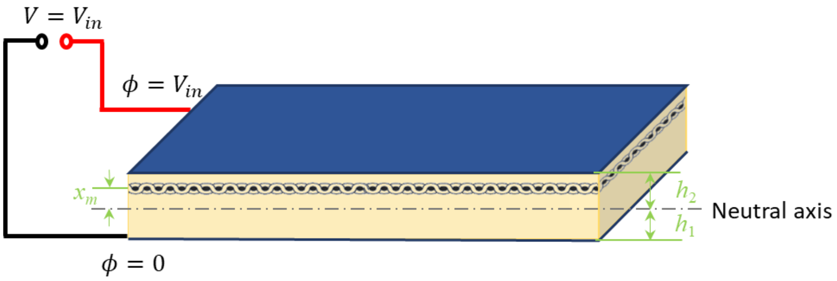

Figure S13. Position of graphene mesh electrode inside the electrolyte membrane.

Substituting c_2 and c_3 in Equation S21, the electric potential along the thickness of actuator is written in terms of c_1 and V_{in} as follows:

$$\phi = -c_1 \cosh(ax) - \left\{\frac{c_1[\cosh(ah_1) - \cosh(ah_2)] - V_{in}}{\sinh(ah_1) + \sinh(ah_2)}\right\} \sinh(ax) + \frac{\sinh(ah_1 + ah_2)}{\sinh(ah_1) + \sinh(ah_2)} c_1 + \frac{\sinh(ah_1)}{\sinh(ah_1) + \sinh(ah_2)} V_{in}$$

$$\phi = c_1\left\{\left(\frac{\cosh(ah_2)-\cosh(ah_1)}{\sinh(ah_1)+\sinh(ah_2)}\right)\sinh ax + \frac{\sinh(ah_1+ah_2)}{\sinh(ah_1)+\sinh(ah_2)} - \cosh(ax)\right\} + \left\{\frac{\sinh(ah_1)+\sinh(ax)}{\sinh(ah_1)+\sinh(ah_2)}\right\}V_{in} \quad \text{(S28)}$$

The absolute potential inside the membrane (V_{sense}) is the output of the measurement at the graphene mesh at $x = x_m$, that is, $\phi(x_m) = V_{sense}$, so that

$$V_{sense} = c_1\left\{\left(\frac{\cosh(ah_2)-\cosh(ah_1)}{\sinh(ah_1)+\sinh(ah_2)}\right)\sinh ax_m + \frac{\sinh(ah_1+ah_2)}{\sinh(ah_1)+\sinh(ah_2)} - \cosh(ax_m)\right\} + \left\{\frac{\sinh(ah_1)+\sinh(ax_m)}{\sinh(ah_1)+\sinh(ah_2)}\right\}V_{in} \quad \text{(S29)}$$

Solving Equation S29 for c_1 yields

$$c_1 = \left\{V_{sense} - \left(\frac{\sinh(ah_1)+\sinh(ax_m)}{\sinh(ah_1)+\sinh(ah_2)}\right)V_{in}\right\} / \left\{\left(\frac{\cosh(ah_2)-\cosh(ah_1)}{\sinh(ah_1)+\sinh(ah_2)}\right)\sinh(ax_m) + \frac{\sinh(ah_1+ah_2)}{\sinh(ah_1)+\sinh(ah_2)} - \cosh(ax_m)\right\}$$

$$c_1 = \frac{1}{\beta}\{[\sinh(ah_1)+\sinh(ah_2)]V_{sense} - [\sinh(ah_1)+\sinh(ax_m)]V_{in}\} \quad \text{(S30)}$$

where,

$$\beta = [\cosh(ah_2)-\cosh(ah_1)]\sinh(ax_m) - [\sinh(ah_1)+\sinh(ah_2)]\cosh(ax_m) + \sinh(ah_1+ah_2) \quad \text{(S31)}$$

Substituting c_1 in Equation S26, the unknown c_2 could be obtain as follows:

$$c_2 = \frac{1}{\beta}\{[\sinh(ah_1) + \sinh(ah_2)]V_{sense} - [\sinh(ah_1) + \sinh(ax_m)]V_{in}\}\frac{\cosh(ah_1) - \cosh(ah_2)}{\sinh(ah_1) + \sinh(ah_2)} - \frac{V_{in}}{\sinh(ah_1) + \sinh(ah_2)}$$

$$c_2 = \left\{\frac{[\sinh(ah_1) + \sinh(ah_2)][\cosh(ah_1) - \cosh(ah_2)]}{\beta[\sinh(ah_1) + \sinh(ah_2)]}\right\}V_{sense} - \left\{\frac{[\sinh(ah_1) + \sinh(ax_m)][\cosh(ah_1) - \cosh(ah_2)] + \beta}{\beta[\sinh(ah_1) + \sinh(ah_2)]}\right\}V_{in} \tag{S32}$$

Within the framework of Nemat Nasser and Li, the internal stress is assumed to be linearly proportional to the charge density [19]. Using this hypothesis, the stress profile along the thickness of the membrane is

$$\sigma = k_0\rho \tag{S33}$$

where σ is the axial stress and k_0 is a gain to be determined from experiments. By substituting ρ from Equation S20 into Equation S33, the induced stress is obtained as follows:

$$\sigma = k_0\kappa_e[c_1a^2\cosh(ax) + c_2a^2\sinh(ax)] \tag{S34}$$

Accordingly, the bending moment generated by ion distribution can be calculated as follows:

$$M = \int_{-h_1}^{h_2} xw\sigma\,dx = \int_{-h_1}^{h_2} wa^2k_0\kappa_e x[c_1\cosh(ax) + c_2\sinh(ax)]\ dx$$

$$M = wa^2k_0\kappa_e\left\{x\left[\frac{c_1}{a}\sinh(ax) + \frac{c_2}{a}\cosh(ax)\right] - \left[\frac{c_1}{a^2}\cosh(ax) + \frac{c_2}{a^2}\sinh(ax)\right]\right\}\Big|_{-h_1}^{h_2}$$

$$M = wk_0\kappa_e\{c_1[ah_2\sinh(ah_2) + ah_1\sinh(ah_1) - \cosh(ah_2) + \cosh(ah_1)] + c_2[ah_2\cosh(ah_2) - ah_1\cosh(ah_1) - \sinh(ah_2) - \sinh(ah_1)]\} \tag{S35}$$

where, w is the width of the actuator. By defining the constant parameters α_1 and α_2 as follows, Equation S35 could be rewritten as a simple form, namely,

$$M = \alpha_1 c_1 + \alpha_2 c_2 \tag{S36}$$

where,

$$\alpha_1 = wk_0\kappa_e[ah_2 \sinh(ah_2) + ah_1 \sinh(ah_1) - \cosh(ah_2) + \cosh(ah_1)] \tag{S37}$$

$$\alpha_2 = wk_0\kappa_e[ah_2 \cosh(ah_2) - ah_1 \cosh(ah_1) - \sinh(ah_2) - \sinh(ah_1)] \tag{S38}$$

By substituting c_1 and c_2 from Equation S30 and S32 into Equation S36, one can obtain the bending moment in terms of V_{sense} and V_{in} :

$$M = \alpha_1 \left\{ \frac{1}{\beta} [\sinh(ah_1) + \sinh(ah_2)] V_{sense} - \frac{1}{\beta} [\sinh(ah_1) + \sinh(ax_m)] V_{in} \right\}$$

$$+ \alpha_2 \left\{ \left[\frac{[\sinh(ah_1) + \sinh(ah_2)][\cosh(ah_1) - \cosh(ah_2)]}{\beta[\sinh(ah_1) + \sinh(ah_2)]} \right] V_{sense} \right.$$

$$\left. - \left[\frac{[\sinh(ah_1) + \sinh(ax_m)][\cosh(ah_1) - \cosh(ah_2)] + \beta}{\beta[\sinh(ah_1) + \sinh(ah_2)]} \right] V_{in} \right\}$$

$$M =$$

$$\left[\frac{[\sinh(ah_1) + \sinh(ah_2)][\alpha_1 \sinh(ah_1) + \alpha_1 \sinh(ah_2) + \alpha_2 \cosh(ah_1) - \alpha_2 \cosh(ah_2)]}{\beta[\sinh(ah_1) + \sinh(ah_2)]} \right] V_{sense}$$

$$-$$

$$\left[\frac{[\sinh(ah_1) + \sinh(ax_m)][\alpha_1 \sinh(ah_1) + \alpha_1 \sinh(ah_2) + \alpha_2 \cosh(ah_1) - \alpha_2 \cosh(ah_2)] + \beta}{\beta[\sinh(ah_1) + \sinh(ah_2)]} \right] V_{in}$$

(S39)

Considering the coefficients of V_{sense} and V_{in} in Equation S39 to be given, the bending moment could be reduced to the simple following form:

$$M = \xi_1 V_{sence} - \xi_2 V_{in} \tag{S40}$$

Where,

$$\xi_1 = \frac{[\sinh(ah_1) + \sinh(ah_2)][\alpha_1 \sinh(ah_1) + \alpha_1 \sinh(ah_2) + \alpha_2 \cosh(ah_1) - \alpha_2 \cosh(ah_2)]}{\beta[\sinh(ah_1) + \sinh(ah_2)]}$$

(S41)

$$\xi_2 = \frac{[\sinh(ah_1) + \sinh(ax_m)][\alpha_1 \sinh(ah_1) + \alpha_1 \sinh(ah_2) + \alpha_2 \cosh(ah_1) - \alpha_2 \cosh(ah_2)] + \beta}{\beta[\sinh(ah_1) + \sinh(ah_2)]}$$

(S42)

Considering the actuator as a cantilever beam, the tip deflection can be calculated as:

$$\delta = \frac{Ml^2}{2E_y I} = \frac{6Ml^2}{wh^3 E_y} \quad \text{(S43)}$$

where, l is the free length of the actuator, E_y is the Young's modulus of the ionic polymer, h is the total thickness of the actuator, and I is the cross-sectional moment of inertia. By substituting the bending moment from Equation S39 into Equation S44, one can obtain the tip deflection induced by ion migration inside the membrane as follows

$$\delta = \frac{6l^2\xi_1}{wh^3E_y}\left(V_{sense} - \frac{\xi_2}{\xi_1}V_{in}\right) \quad \text{(S44)}$$

The obtained equation is similar to the Static Model proposed in the main text:

$$\delta_{SM} = C_2(V_{sense} - C_1 V_{in}) \quad \text{(S45)}$$

Phase Delay Modification for Dynamic Model

The phase delay for dynamic inputs can be adjusted using the following equation:

$$\sin(\omega t + \varphi) = \sin\omega t \cos\varphi + \cos\omega t \sin\varphi \quad \text{(S46)}$$

Considering $\cos\omega t = \frac{1}{\omega}\frac{d}{dt}(\sin\omega t)$, one can obtain

$$\sin(\omega t + \varphi) = \sin\omega t \cos\varphi + \left[\frac{1}{\omega}\frac{d}{dt}(\sin\omega t)\right]\sin\varphi \quad \text{(S47)}$$

By supposing that the signal obtained from the static model is a harmonic signal ($\sin\omega t$), Equation S47 can be used to obtain the shifted signal of static model as follows:

$$\delta_{DM} = \delta_{SM} \cos \varphi + \frac{1}{\omega} \delta_{SM}' \sin \varphi \tag{S48}$$

where δ_{DM} is the signal for the dynamic model and has φ radian phase difference with δ_{SM}.

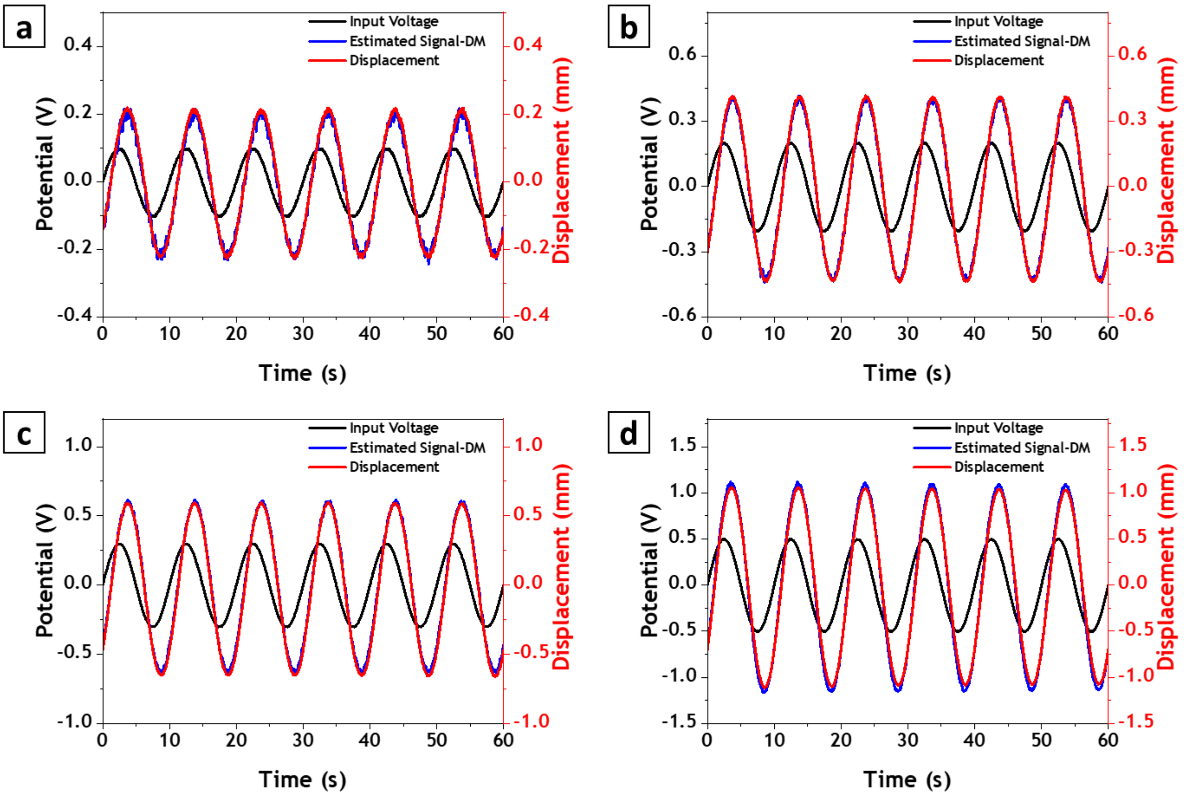

Figure S14. Estimation of response of self-sensing actuator to AC stimulation using Dynamic Model: a) 0.1 V sine wave input, b) 0.2 V sine wave input, c) 0.3 V sine wave input d) 0.5 V sine wave input.

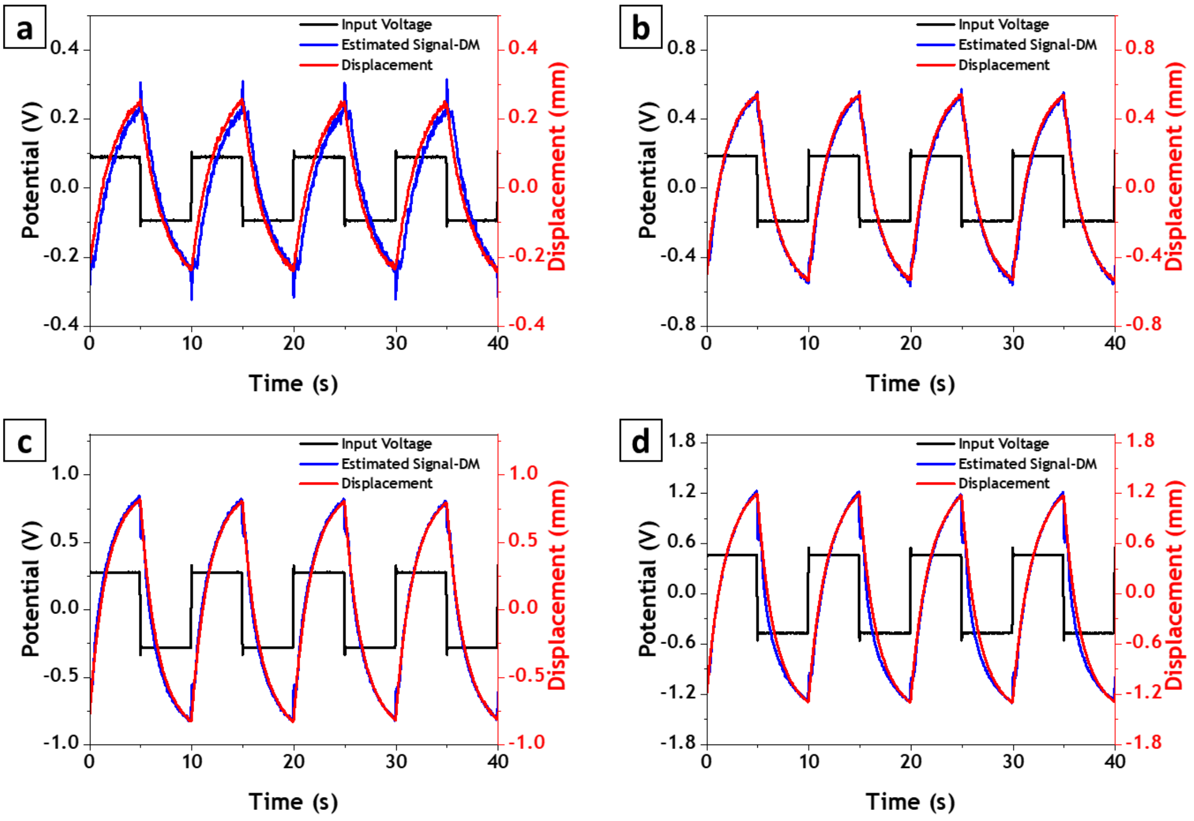

Figure S15. Estimating response of self-sensing actuator to square wave stimulation using Dynamic Model: a) 0.1 V square wave input, b) 0.2 V square wave input, c) 0.3 V square wave input d) 0.5 V square wave input.

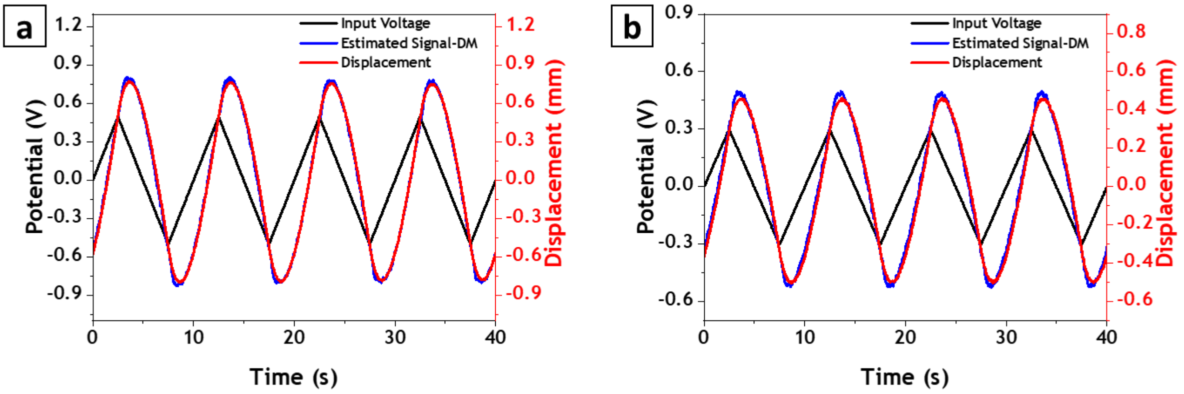

Figure S16. Estimating response of self-sensing actuator to triangle wave stimulation using Dynamic Model: a) 0.3 V triangle wave input and b) 0.5 V triangle wave input.

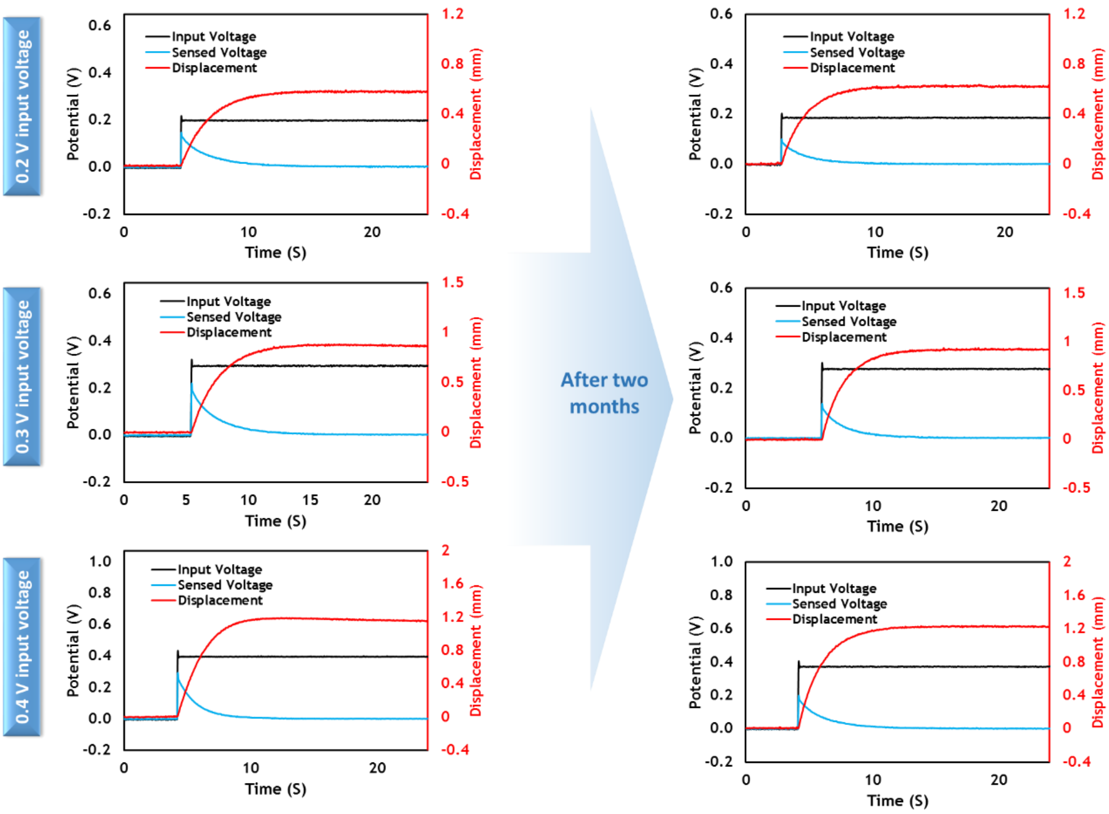

Figure S17. Checking the performance of the self-sensing actuator after two months.
